# Tri-channel synergistic model for 3D human pose estimation

**DOI:** 10.1371/journal.pone.0359200

**Published:** 2026-10-15

**Authors:** Ying Gu, Chenglong Li, Xinyan Gao, Jinxiao Zhang

**Affiliations:** 1 Shandong Jianzhu University, Jinan, Shandong Province, China; 2 Shandong Huayun 3D Technology Co., Ltd., Jinan, Shandong Province, China; Nanchang University, CHINA

## Abstract

3D human pose estimation from single-view images is a fundamental and challenging task in computer vision, the key of which lies in effectively capturing the complex spatial dependencies between human joint points. Existing methods usually rely on a single computational paradigm: Graph Convolutional Networks (GCN) excel at capturing local physical constraints, the self-attention mechanism (Attn) is adept at modeling global dynamic correlations, and Multi-Layer Perceptrons (MLP) provide an efficient way for global context encoding. Although previous works have attempted to fuse these methods, they often adopt serial, simple parallel or dual-stream structures, failing to fully explore the synergistic potential among these three heterogeneous computational paradigms, which results in limited representation capability under complex or rare poses. To address the above limitations, this paper proposes a novel Tri-Channel Synergistic Model (TCSM) aiming to construct a more comprehensive and robust feature representation for single-frame pose estimation. The core idea of this model is to decouple the dependencies of different natures in human poses and perform optimal parsing in independent and functionally specific channels. Specifically, our model designs three parallel feature streams: a GCN channel for encoding structure modality based on human topology, an attention channel for capturing long-range dynamic correlation modality, and an MLP channel for efficiently extracting global context modality. To achieve deep synergy, we design a two-stage feature fusion and refinement mechanism, in which the outputs of each channel interact and are iteratively enhanced. Experiments on the Human3.6M benchmark demonstrate that TCSM achieves competitive single-frame performance. The model obtains 47.7 mm MPJPE and 37.8 mm PA-MPJPE without a pose refinement module, and the ablation results verify the contribution of the tri-channel design and the two-stage interaction mechanism.

## Introduction

3D Human Pose Estimation (3DHPE) is a fundamental task in the field of computer vision, aiming to recover the 3D spatial coordinates of human skeletal joints from 2D observation data such as images or videos. Due to its core role in numerous downstream applications including human-computer interaction, virtual/augmented reality (VR/AR), sports biomechanical analysis, intelligent surveillance and autonomous driving, this field has received extensive attention from academia and industry in recent years [1,2]. Accurate 3D pose information can provide key geometric clues for machines to understand human behaviors, intentions and states, and is an important cornerstone for achieving deeper human-centric AI [3,4].

At present, the two-stage method based on “2D-to-3D Lifting” has become the mainstream paradigm for 3DHPE [5]. This paradigm first uses a mature high-precision 2D pose detector to extract joint coordinates from images, and then designs a lightweight network to map these 2D coordinates to 3D space. This method effectively avoids the huge complexity of end-to-end learning of 3D poses directly from image pixels. However, this lifting process is essentially an ill-posed problem, because a single 2D projection may correspond to an infinite number of valid 3D poses, that is, there is inherent depth ambiguity. Therefore, the key to inferring a unique and accurate 3D structure from limited 2D input lies in whether the network can effectively capture and utilize the complex intrinsic dependencies between human joint points [6,7].

To parse these dependencies, existing works have mainly explored three computational paradigms. First, GCN-based methods regard the human skeleton as a graph structure and use its topological prior to capture local physical constraints between joint points [8–10]. Such methods perform excellently in characterizing kinematic chains, but their limited receptive field makes it difficult to capture global correlations between distant joint points. Second, Transformer models based on Attn can effectively capture long-range, dynamic dependencies between any two joint points by virtue of their powerful global information interaction capability [5,11–13]. However, such methods usually have high computational costs, and sometimes produce physically unreasonable predictions due to the lack of explicit structural priors. Recently, inspired by the MLP-Mixer architecture, MLP-based [14,15] methods have emerged as an efficient alternative, which can obtain global context information with low computational overhead, but may not match Attn in the accuracy of parsing complex dynamic correlations.

Although previous works have attempted to fuse the above paradigms, such as adopting dual-stream structures or interleaving mechanisms, they still have certain limitations. These methods fail to systematically decouple and synergize the dependencies of different natures in human poses—namely, physics-based structural modality, data-driven dynamic correlation modality and efficient global context modality. We believe that coupling these heterogeneous dependencies in a single or dual processing stream will limit the comprehensiveness and robustness of model representation [6,7].

To address the above challenges, this paper proposes a novel TCSM, a parallel fusion model specially designed for single-frame 3D human pose estimation. The core idea of our work is “divide and conquer”: decoupling the complex pose dependency parsing task into three parallel and functionally specific channels. Specifically, a GCN channel is responsible for parsing local structural constraints, an attention channel focuses on capturing long-range dynamic correlations, and an MLP channel efficiently extracts global context. To achieve deep synergy among these three heterogeneous feature streams, we design a two-stage feature fusion and refinement mechanism, in which the outputs of each channel fully interact in the intermediate stage, and the fused enhanced information is used to guide the feature refinement in the second stage.

The main contributions of this paper can be summarized as follows:

- We propose a novel tri-channel parallel architecture that decouples and synergistically uses three computational paradigms (GCN, Attn and MLP) for the first time to parse human pose dependencies of different natures.
- We design a two-stage feature fusion and refinement mechanism to realize deep information interaction and iterative enhancement among three heterogeneous feature streams.

## Related work

### A. GCN-based methods

The natural topological structure of the human skeleton makes it very suitable for representation by a graph, where joint points are the vertices of the graph and bones are the edges. Therefore, GCN are widely used in 3D human pose estimation tasks [16,17]. The core advantage of such methods is that they can explicitly use human kinematic priors and effectively capture local physical constraints and dependencies by aggregating features of neighboring joint points. For example, Cai et al. used spatiotemporal GCN to explore the relationships between joint points [8]; Zou et al. proposed MGCN which enhanced model performance through modulated graph convolution [9]; Yu et al. proposed GLA-GCN to improve the performance of 3D human pose lifting based on ground-truth 2D poses by globally modeling spatiotemporal graph structures and locally backtracking joint features [10]. However, the inherent limitation of GCN is its limited receptive field, which is usually only limited to first-order or second-order neighborhoods. This makes it difficult to capture global correlations between distant joint points, such as the coordination between hands and feet in complex movements such as running or dancing.

### B. Attn-based methods

To overcome the deficiency of GCN in capturing global information, researchers have introduced the Transformer architecture which has achieved great success in the field of natural language processing, the core of which is Attn [18]. Attn-based methods regard the human joint point sequence as a set of tokens, and can flexibly capture long-range, dynamic dependencies between any two joint points by calculating the correlation weights between all token pairs [19,20]. PoseFormer proposed by Zheng et al. is a typical example, which demonstrates the powerful capability of spatiotemporal Transformer in this task [5]; Tang et al. proposed STCFormer to efficiently model the spatiotemporal dependencies of human joints in videos and improve the accuracy of 3D pose estimation through spatiotemporal cross attention and structure-enhanced position embedding [11]. Although Attn performs excellently in capturing global information, it also has two main problems: first, its computational cost with quadratic complexity is relatively high; second, it ignores the inherent skeletal topological structure of the human body in the processing process, and sometimes may produce physically unreasonable prediction results [12,13].

### C. MLP-based methods

In recent years, inspired by works such as MLP-Mixer, pure MLP architectures have emerged as an efficient alternative in the field of computer vision [14]. Such methods only use simple MLP acting alternately on the spatial (inter-token) and channel (intra-token) dimensions, and can achieve a global receptive field with low computational overhead [14]. Li et al. first introduced this idea into the field of 3D human pose estimation and proposed GraphMLP [15,21]. They found that although the original MLP architecture is efficient, it is insufficient in capturing fine local details and fusing human structural priors [13,22]. Therefore, GraphMLP proved the effectiveness of explicitly injecting graph structure information into the MLP framework by combining GCN and MLP in parallel [7,15].

### D. Hybrid models

To combine the advantages of different paradigms, designing hybrid models has become a current research trend [1]. Existing fusion strategies can be mainly divided into several categories: parallel fusion, such as GraphMLP which directly adds the outputs of GCN and MLP; serial or interleaved fusion, such as IGANet [6] which designed a bidirectional guidance mechanism (G2A and A2G) between GCN and Attn to achieve deep coupling of the two modules; and parallel stream fusion, such as DC-GCT which places GCN and Attn in two independent parallel processing chains and performs information interaction through a central module.In addition to hybrid architectural design, Peng et al. proposed a dual-augmentor framework for domain generalization in 3D human pose estimation, which improves the robustness of pose estimators under cross-domain variations [23]. Peng et al. further investigated lifelong domain adaptive 3D human pose estimation, aiming to adapt pose estimation models to continuously changing target domains while alleviating catastrophic forgetting [24]. These works have greatly promoted the development of the field and proved the huge potential of fusing different computational paradigms. However, they are usually limited to the combination of two paradigms. We believe that GCN, Attn and MLP, these three powerful building blocks, have irreplaceable advantages in capturing physical structure, dynamic correlation and efficient context respectively. How to systematically decouple and synergize these three heterogeneous computational paradigms within a unified framework remains an open question [24]. Inspired by this, our work aims to explore this direction. By designing a tri-channel parallel architecture, each of the three paradigms performs its own duties, and deep synergy is achieved through an iterative refinement mechanism, thus providing a more comprehensive and robust solution for 3D human pose estimation [25].

## Methodology

To solve the limitations of existing methods that rely solely on graph convolution or Transformer, we propose a novel TCSM. The core of TCSM is a parallel processing architecture that integrates three heterogeneous feature extraction mechanisms in a unified module: GCN for capturing skeletal topology, Attn for modeling global dependencies, and MLP for abstract feature encoding. In addition, we design a Dual-Stage Interaction Strategy that allows these heterogeneous features to perform intensive interaction and correction along the depth direction of the network.

### A. Overall architecture

The overall architecture of our model is shown in Fig 1, which follows a standard “encoder-decoder” paradigm [26,27]. The model consists of three main stages: an initial feature encoding stage, a deep feature extraction stage composed of stacked tri-channel modules, and a final output projection stage. The overall design concept is to integrate the complementary advantages of different neural network paradigms through a unified collaborative framework—GCN for local structure capture, Attn for processing global dependency relationships, and MLP for efficient feature fusion. This multi-dimensional method enables the model to capture richer and more robust human pose representations, effectively breaking through the limitations of single feature extractor methods. The architecture combines strong performance and efficiency, and the clear division of responsibilities among each component not only ensures interpretability but also achieves modular design.

**Fig 1:**
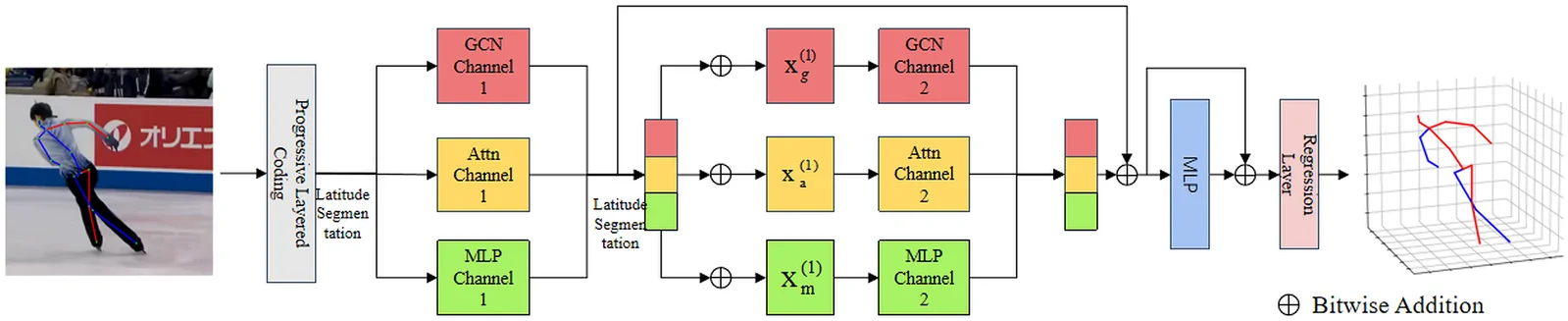
Overall model architecture diagram (This figure was created by the authors).

### B. Progressive feature embedding

Traditional Lifting methods usually use a single-layer linear projection to map 2D coordinates to a high-dimensional space, which may lead to premature loss of spatial information [5,27]. On the contrary, we adopt a Progressive Encoder to gradually enhance the feature dimension. Let ${\text{X}}_{\text{in}}\in R^{\text{B}\times \text{J}\times \text{2}}$ be the input 2D coordinates, we construct the mapping through stacked linear layers:


$$\begin{array}{c} \begin{array}{c} {\text{Z}}_{\text{0}}={\text{E}}_{\text{prog}}({\text{X}}_{\text{in}})+{\text{E}}_{\text{pos}} \end{array} \end{array}$$ (1)

Where$\;{\text{E}}_{\text{prog}}(\cdot )$ represents an MLP network with gradually increasing dimensions of 2 → 64 → 128 → C, and ${\text{E}}_{\text{pos}}\in R^{\text{1}\times \text{J}\times \text{C}}$ is a learnable spatial position encoding used to retain the identity information of joint points. ${\text{Z}}_{\text{0}}\in R^{\text{B}\times \text{J}\times \text{C}}$ will be used as the initial feature input to the subsequent tri-channel modules, where B is the batch size, J is the number of joints, and C is the number of channel dimensions of the model. The number of joints in this paper is 17.

### C. Tri-channel synergistic module

This is the core component of our model. For the input feature ${\text{Z}}_{\text{l}-\text{1}}$ of the l-th layer, we first split it into three sub-features along the channel dimension: ${\text{Z}}_{\text{gcn}},{\text{Z}}_{\text{attn}}\text{ and }{\text{Z}}_{\text{mlp}}$, corresponding to topological, global and semantic features respectively. These features are sent to three parallel branches for processing, and the structure diagram of the three channels is shown in Fig 2.

**Fig 2:**
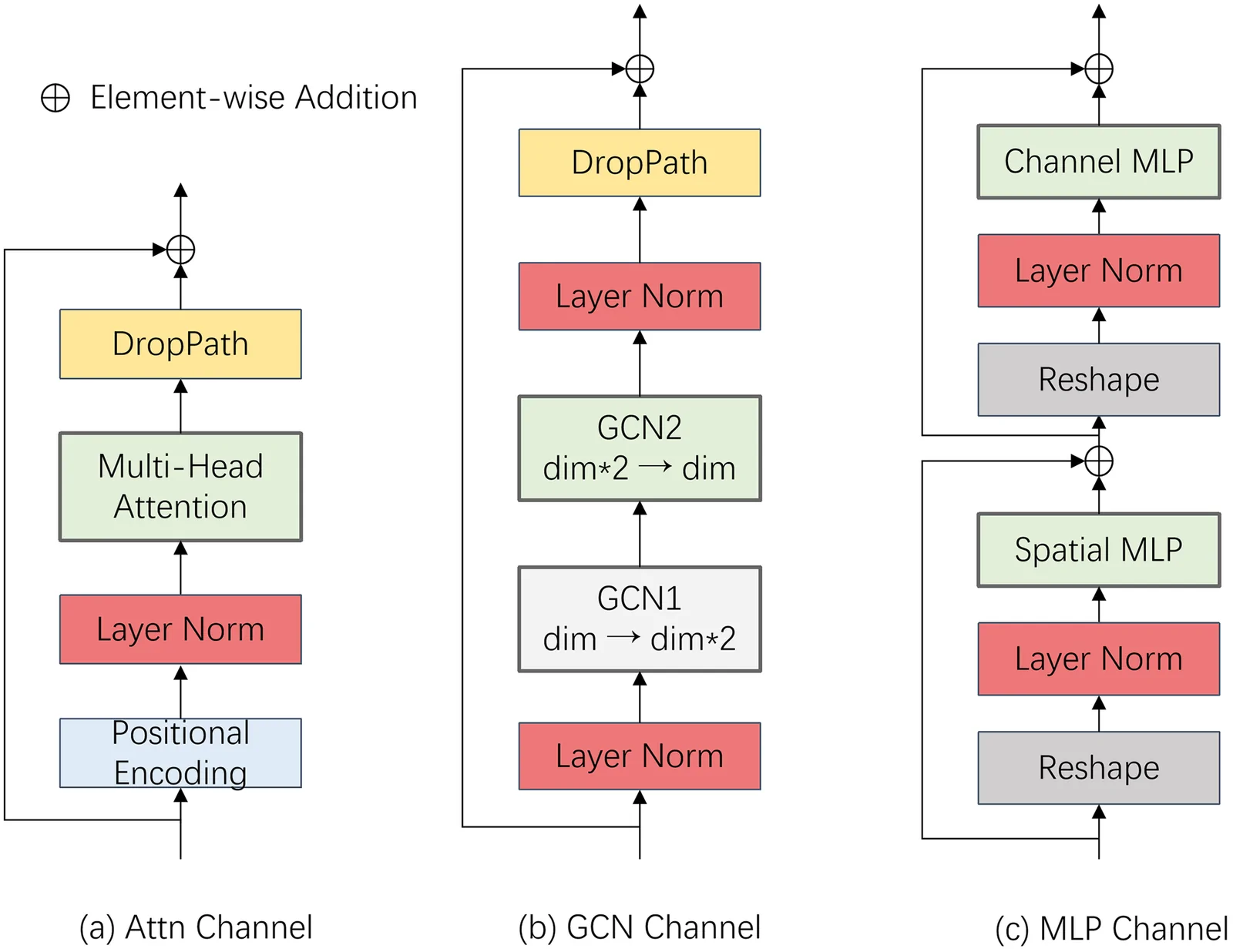
Three-channel structure diagram. (This figure was created by the authors).

#### 1. Attn channel.

The core of the Attn channel is the Attention module, which implements the scaled dot-product attention mechanism [5,11,12]. Given an input feature tensor ${\text{X}}_{\text{attn}}$ with the shape of (B*F, J, dim_attn), the module first generates three linear projections: Query (Q), Key (K), and Value (V). These projections are obtained by applying learnable weight matrices to the input features. Then the attention scores are obtained by calculating the dot product of the query tensor and the key tensor, the result is multiplied by the square root of the feature dimension to prevent the softmax function from saturation, and the softmax function is applied to obtain the probability distribution of joints. These attention scores represent the importance of each joint feature to all other joints. The final output is calculated by weighted summation of the value tensor using the attention scores as weights. This process is applied in parallel to h “heads” (num_heads), and each head learns to focus on different types of feature relationships or subspaces. The definition of a single head is as follows:


$$\begin{array}{c} \begin{array}{c} {\text{head}}_{\text{i}}=\text{softmax}\left(\frac{({\text{X}}_{\text{attn}}{\text{W}}_{\text{i}}^{\text{Q}})({\text{X}}_{\text{attn}}{\text{W}}_{\text{i}}^{\text{K}}{)}^{\text{T}}}{\sqrt{{\text{d}}_{\text{k}}}}\right)({\text{X}}_{\text{attn}}{\text{W}}_{\text{i}}^{\text{V}}) \end{array} \end{array}$$ (2)

Where ${\text{W}}_{\text{i}}^{\text{Q}}{,\text{W}}_{\text{i}}^{\text{K}},{\text{W}}_{\text{i}}^{\text{V}}$ are the query, key and value transformation matrices of the i head respectively, and ${\text{d}}_{\text{k}}$ is the dimension of a single head. The outputs of all heads are then concatenated and projected back to the original dimension:


$$\begin{array}{c} \begin{array}{c} {\text{X}}_{\text{attn}\_\text{out}}=\text{Concat}([{\text{head}}_{\text{1}},\cdots ,{\text{head}}_{\text{h}}]){\text{W}}^{\text{O}} \end{array} \end{array}$$ (3)

Where ${\text{W}}^{\text{O}}$ is a linear projection matrix that mixes the multi-head attention outputs, integrating the features of each head into a unified final representation, and then a residual connection is applied to prevent excessive feature smoothing. This multi-head design enables the model to jointly pay attention to information from different representation subspaces at different positions, significantly enhancing its representation capability. This allows the model to break through the limitations of graph structure and adaptively focus on global motion patterns.

#### 2. GCN channel.

The design of the GCN channel aims to explicitly model the local spatial relationships between human joints, which are defined by the physical structure of the skeleton [8–10]. This channel applies the principles of GCN, a type of network specially suitable for processing data that can be represented as graph structures. In the context of human pose estimation, joints are regarded as nodes in the graph, and the bones connecting them serve as edges. By performing convolution operations on this graph structure, the GCN channel can effectively aggregate information from neighboring joints, thereby learning features that conform to human anatomical characteristics. The GCN channel consists of two consecutive GCN blocks, namely gcn1 and gcn2, which together form a residual unit. The first block gcn1 receives input features with dimension dim_gcn and transforms them into a higher-dimensional space with dimension dim_gcn * 2. This feature expansion enables the model to learn richer representations in the middle layer. The first layer can be expressed as:


$$\begin{array}{c} \begin{array}{c} {\text{X}}_{\text{mid}}=\sigma \left(\text{LN}\left(\sum_{\text{k}=\text{1}}^{\text{K}}\;{\text{A}}_{\text{k}}{\text{X}}_{\text{gcn}}{\text{W}}_{\text{k}}^{\left(\text{1}\right)}\right)\right) \end{array} \end{array}$$ (4)

where σ(·) is the GELU activation function, introducing complex interaction capabilities. LN(·) is LayerNorm layer normalization. ${\text{A}}_{\text{k}}$ is the adjacency matrix of the k-th subgraph with pre-defined skeleton connections. ${\text{X}}_{\text{gcn}}$ is the input feature of the GCN layer. ${\text{W}}_{\text{k}}^{\left(\text{1}\right)}$ is the learnable weight matrix of the first layer of the k-th subgraph, realizing dimension-up transformation of features and capturing local spatial patterns.

The second block gcn2 maps these intermediate features back to the original dimension dim_gcn. The second layer of the GCN channel can be expressed as:


$$\begin{array}{c} \begin{array}{c} {\text{X}}_{\text{gc}{\text{n}}_{\_}\text{out}}=\sum_{\text{k}=\text{1}}^{\text{K}}\;{\text{A}}_{\text{k}}{\text{X}}_{\text{mid}}{\text{W}}_{\text{k}}^{\left(\text{2}\right)} \end{array} \end{array}$$ (5)

where ${\text{W}}_{\text{k}}^{\left(\text{2}\right)}$ is the learnable weight matrix of the second layer of the k-th subgraph, realizing dimension-down compression of features and extracting core topological features.

This design helps reduce the number of parameters while maintaining model capacity. The GCN channel is implemented through a residual block containing two GCN layers, making it able to learn complex nonlinear transformations of skeletal features while maintaining the stability of gradients during training.

#### 3. MLP channel.

As the third core module of the feature extraction strategy, the MLP channel realizes information fusion of spatial and channel dimensions through a computationally efficient mechanism [14,15]. Different from the GCN channel focusing on local structure and the attention channel capturing global dependency relationships, the MLP channel follows the principle of feature mixing, and its architecture is similar to models such as MLP-Mixer. This channel consists of two core components: Spatial MLP and Channel MLP. Spatial MLP enables each channel to fuse information across joints, helping the model learn the inter-joint correlations not explicitly defined by the skeleton graph or global attention mechanism. Its process can be expressed as:


$$\begin{array}{c} \begin{array}{c} \text{Z}={\text{X}}_{\text{MLP}}+\text{DropPath}({\text{Linear}}_{\text{2}}(\sigma ({\text{Linear}}_{\text{1}}({\text{LN}}_{\text{J}}({\text{X}}_{\text{MLP}}))))) \end{array} \end{array}$$ (6)

Where ${\text{X}}_{\text{MLP}}$ is the input feature of the Spatial MLP layer, and the number of joints is regarded as the feature dimension after shape rearrangement. ${\text{LN}}_{\text{J}}(\cdot )$ is normalized along the joint dimension, different from the standard LayerNorm. ${\text{Linear}}_{\text{1}}$ and ${\text{Linear}}_{\text{2}}$ are dimension-up and dimension-down operations respectively, aiming to learn inter-joint combinations and then map the features back to the original space. σ(·) is the GELU activation function.Channel MLP allows each joint to integrate information from all its feature channels, realizing complex nonlinear transformation of joint representations. Its process can be expressed as:


$$\begin{array}{c} \begin{array}{c} {\text{X}}_{\text{MLP}\_\text{out}}={\text{X}}_{\text{mid}}+\text{DropPath}({\text{Linear}}_{\text{4}}(\sigma ({\text{Linear}}_{\text{3}}({\text{LN}}_{{\text{D}}_{\text{mip}}}({\text{X}}_{\text{mid}}))))) \end{array} \end{array}$$ (7)

where ${\text{X}}_{\text{mid}}$ is the input tensor of Channel MLP, recovered by rearranging the shape of ${\text{LN}}_{{\text{D}}_{\text{mlp}}}(\cdot )$ is the standard layer normalization along the feature dimension, calculating the mean and variance of the ${\text{D}}_{\text{mlp}}$ dimensional features independently for each joint to stabilize the feature distribution. ${\text{Linear}}_{\text{3}}$ and ${\text{Linear}}_{\text{4}}$ are dimension-up and dimension-down operations respectively, to increase the capacity of feature channels, and screen and compress high-dimensional information to retain key features. σ(·) is the GELU activation function.

This dual mixing design enables the MLP channel to process feature tensors in an integrated manner, forming a complement to the specialized operations of the GCN channel and attention channel. The MLP channel adopts a residual block structure combined with layer normalization technology to ensure stable and efficient training process.

#### 4. Two-stage interactive fusion.

To avoid the isolated feature learning of different branches, we propose a residual-based two-stage fusion mechanism. This mechanism is crucial for exerting the synergistic advantages of the tri-channel design [1,6,7], because it allows each channel to learn from the features learned by other channels. The fusion process is designed with both strong performance and efficiency, and effectively integrates different feature representations through a combination of series connection, learnable MLP and residual connection.

In the first stage, the input feature tensor is split according to the dimension allocation strategy, and the three generated sub-tensors are independently processed by the corresponding channels: gcn_channel_1, attn_channel_1 and mlp_channel_1. This enables each channel to perform its specialized feature extraction on the initial representation. The outputs of these first-round channels are then concatenated to form a single fused feature tensor ${\text{X}}_{\text{cat}}$. This tensor is then processed by fusion_mlp—a small MLP serving as a learnable fusion mechanism that enables the model to weight and combine information from different channels. The output of fusion_mlp is added back to ${\text{X}}_{\text{cat}}$ through residual connection to generate the intermediate fusion representation ${\text{X}}_{\text{cat}}$. The process can be expressed as follows:


$$\begin{array}{c} \begin{array}{c} \left({\text{X}}_{\text{g}},{\text{X}}_{\text{a}},{\text{X}}_{\text{m})}=\text{Split}(\text{X}\right) \end{array} \end{array}$$ (8)

$$\begin{array}{c} \begin{array}{c} \left\{ \begin{array}{cccc} & {\text{X}}_{\text{g}}^{\left(\text{1}\right)}={\text{F}}_{\text{gcn}}({\text{X}}_{\text{g}}) & & \\ & {\text{X}}_{\text{a}}^{\left(\text{1}\right)}={\text{F}}_{\text{attn}}({\text{X}}_{\text{a}}) & & \end{array}\right. \end{array} \end{array}$$ (9)

$$\begin{array}{c} \begin{array}{c} {\text{X}}_{\text{cat}}=\text{Concat}\left([{\text{X}}_{\text{g}}^{\left(\text{1}\right)},{\text{X}}_{\text{a}}^{\left(\text{1}\right)},{\text{X}}_{\text{m}}^{\left(\text{1}\right)}]\right) \end{array} \end{array}$$ (10)

$$\begin{array}{c} \begin{array}{c} {\text{X}}_{\text{fusion}}={\text{X}}_{\text{cat}}+\text{MLP}\left(\text{LN}({\text{X}}_{\text{cat}})\right) \end{array} \end{array}$$ (11)

where X is the total feature output by the encoder, ${\text{X}}_{\text{g}},{\text{X}}_{\text{a}},{\text{X}}_{\text{m}}$ are the inputs corresponding to gcn_channel_1, attn_channel_1 and mlp_channel_1 respectively, and the outputs obtained after independent processing by each channel are ${\text{x}}_{\text{g}}^{\left(\text{1}\right)}$, ${\text{x}}_{\text{a}}^{\left(\text{1}\right)}$,${\text{x}}_{\text{m}}^{\left(\text{1}\right)}$. Concat is the feature concatenation operation.

In the second stage, the fused representation is split again into three parts. The key point is that these re-split features are no longer processed independently, but are added to the outputs of the first-stage channels before being input to the second-round channels (gcn_channel_2, attn_channel_2, mlp_channel_2). This cross-channel information flow allows each second-stage channel to refine its features using complementary information obtained from the other channels in the first stage.This cross-channel information flow is the core advantage of this module: each channel in the second stage can optimize its own features based on the information collected by other channels in the first stage. For example, when optimizing local spatial features, the second GCN channel can integrate the global context information provided by the attention channel and the mixed features of the MLP channel. This two-stage cross-feeding design produces a strong synergistic effect, enabling the model to learn highly complex and interdependent feature representations. The process can be expressed as follows:


$$\begin{array}{c} \begin{array}{c} {\text{X}}_{\text{fg}},{\text{X}}_{\text{fa}},{\text{X}}_{\text{fm}}=\text{Split}\left({\text{X}}_{\text{fusion}}\right) \end{array} \end{array}$$ (12)

$$\begin{array}{c} \begin{array}{c} \left\{ \begin{array}{cc} {\text{X}}_{\text{g}}^{\left(\text{2}\right)}={\text{F}}_{\text{gcn}}\left({\text{X}}_{\text{g}}^{\left(\text{1}\right)}+{\text{X}}_{\text{fg}}\right) & \\ {\text{X}}_{\text{a}}^{\left(\text{2}\right)}={\text{F}}_{\text{attn}}\left({\text{X}}_{\text{a}}^{\left(\text{1}\right)}+{\text{X}}_{\text{fa}}\right) & \\ {\text{X}}_{\text{m}}^{\left(\text{2}\right)}={\text{F}}_{\text{mlp}}\left({\text{X}}_{\text{m}}^{\left(\text{1}\right)}+{\text{X}}_{\text{fm}}\right) & \end{array}\right.\; \end{array} \end{array}$$ (13)

$$\begin{array}{c} \begin{array}{c} {\text{X}}_{\text{final}}=\text{Concat}\left([{\text{X}}_{\text{g}}^{\left(\text{2}\right)},{\text{X}}_{\text{a}}^{\left(\text{2}\right)},{\text{X}}_{\text{m}}^{\left(\text{2}\right)}]\right)+{\text{X}}_{\text{fusion}} \end{array} \end{array}$$ (14)

$$\begin{array}{c} \begin{array}{c} {\text{X}}_{\text{out}}={\text{X}}_{\text{final}}+\text{DropPath}\left({\text{MLP}}_{\text{final}}\left(\text{LN}({\text{X}}_{\text{final}})\right)\right) \end{array} \end{array}$$ (15)

where ${\text{X}}_{\text{g}}^{\left(\text{2}\right)},{\text{X}}_{\text{a}}^{\left(\text{2}\right)},{\text{X}}_{\text{m}}^{\left(\text{2}\right)}$ are the inputs corresponding to gcn_channel_2, attn_channel_2 and mlp_channel_2 respectively, and the outputs obtained after independent processing by each channel are ${\text{x}}_{\text{g}}^{\left(\text{2}\right)}$, ${\text{x}}_{\text{a}}^{\left(\text{2}\right)}$, ${\text{x}}_{\text{m}}^{\left(\text{2}\right)}$. ${\text{X}}_{\text{final}}$ is the final feature of the fusion block, obtained by adding the concatenation result of the second round of the three channels with the intermediate fusion feature ${\text{X}}_{\text{fusion}}$, containing complete multi-scale information. ${\text{X}}_{\text{out}}$ is the final output.

After the second round of processing, the outputs of the three channels are concatenated to form the feature representation ${\text{X}}_{\text{final}}$. This representation then generates the output of the tri-channel module through the final MLP. After the residual operation, the final output result ${\text{X}}_{\text{out}}$ of the module is formed. This final step of fusion and MLP processing ensures that the output of the tri-channel module is a rich and fully processed feature representation that can be directly used for the final 3D coordinate regression.

### D. 3D pose regression

After processing by the tri-channel module, we obtain the final feature representation ${\text{X}}_{\text{out}}$. Our prediction head uses a linear layer for regression [5]. Applying it to the last layer feature ${\text{X}}_{\text{out}}$, we get the final 3D human pose ${\text{X}}_{\text{output}}\in R^{\text{B}\times \text{J}\times \text{3}}$.

### E. Loss function

To train TCFM in an end-to-end manner, we adopt the $L_{\text{2}}-\text{norm}$ loss as the objective function [5]. This loss function calculates the average Euclidean distance between the predicted 3D joint coordinates and the ground truth annotations. Its definition is as follows:


$$\begin{array}{c} \begin{array}{c} L=\frac{\text{1}}{\text{N}}\sum_{\text{i}=\text{1}}^{\text{N}}∥{\hat{\text{J}}}_{\text{i}}-{\text{J}}_{\text{i}}{∥}_{\text{2}} \end{array} \end{array}$$ (16)

where N is the number of joint points on the human body. ${\hat{\text{J}}}_{\text{i}}$ is the 3D coordinate of the i-th joint point predicted by the model. ${\hat{\text{J}}}_{\text{i}}$ is the ground truth 3D coordinate of the i-th joint point. $∥\cdot {∥}_{\text{2}}$ represents the Euclidean distance (L2 norm). We minimize this loss during training to drive the model to learn accurate 3D spatial mapping.

## Experiments

### A. Experimental setup

The experimental platform in this paper uses Windows 10 Home Edition, with an Intel(R) Core(TM) i9-10900X CPU @ 3.70GHz processor and an NVIDIA RTX 3090 GPU. The deep learning framework is pytorch version 2.4.0, and the programming language is Python 3.9. A variety of data augmentation techniques are adopted in the experiments. For optimizing model parameters, we select the Adamw optimizer, set the base learning rate to 1e-3, reduce the learning rate to 0.95 of the previous round after each training epoch, and reduce the learning rate to 0.5 of the previous round every five training epochs. The entire training process is completed within 30 training epochs. We use the Cascade Pyramid Network (CPN) to detect 2D human pose estimation on the Human3.6M dataset [28], and then use the 2D human pose estimation results as the input of the model to complete the entire 3D human pose estimation process.

### B. Dataset description

We used the Human3.6M benchmark dataset, which contains approximately 3.6 million video frames from 11 subjects performing 15 actions and provides synchronized 2D and 3D joint annotations. Following the standard evaluation protocol, subjects S1, S5, S6, S7, and S8 were used for training, and subjects S9 and S11 were used for testing. The model was evaluated using MPJPE and PA-MPJPE. MPJPE is the mean Euclidean distance between predicted and ground-truth 3D joints over all test frames and joints. PA-MPJPE is computed after Procrustes alignment, which removes global translation, rotation, and scale differences and therefore evaluates the relative pose structure.

### C. Quantitative analysis

Table 1 reports in detail the comparison results between the TCSM proposed in this paper and other state-of-the-art methods with single-frame as input on the Human3.6M dataset. It can be seen that TCSM achieves 47.7 mm in MPJPE and 37.8 mm in P-MPJPE, which is superior to previous methods. In addition, some methods adopt a pose refinement module to further improve performance. By integrating such a module, we achieve a lower MPJPE (47.1 mm), with a 2.3 mm error reduction compared with MGCN.

**Table 1:** Experimental results of MPJPE and P-MPJPE on human 3.6M for different single-frame models.

MPJPE	Dir.	Disc	Eat	Greet	Phone	Photo	Pose	Purch.	Sit	SitD.	Smoke	Wait	WalkD.	Walk	WalkT.	Avg.
FCN [25].	51.8	56.2	58.1	59.0	69.5	78.4	55.2	58.1	74.0	94.6	62.3	59.1	65.1	49.5	52.4	62.9
TCN [29]	47.1	50.6	49.0	51.8	53.6	61.4	49.4	47.4	59.3	67.4	52.4	49.5	55.3	39.5	42.7	51.8
ST-GCN [17]	46.5	48.8	47.6	50.9	52.9	61.3	48.3	45.8	59.2	64.4	51.2	48.4	53.5	39.2	41.2	50.6
SRNet [30]	44.5	48.2	47.1	47.8	51.2	56.8	50.1	45.6	59.9	66.4	52.1	45.3	54.2	39.1	40.3	49.9
GroupSH [28]	45.2	49.9	47.5	50.9	54.9	66.1	48.5	46.3	59.7	71.5	51.4	48.6	53.9	39.9	44.1	51.9
MGCN [9]	45.4	49.2	45.7	49.4	50.4	58.2	47.9	46.0	57.5	63.0	49.7	46.6	52.2	38.9	40.8	49.4
GraFormer [31]	45.2	50.8	48.0	50.0	54.9	65.0	48.2	47.1	60.2	70.0	51.6	48.7	54.1	39.7	43.1	51.8
URGN	47.9	50.0	47.1	51.3	51.2	59.5	48.7	46.9	56.0	61.9	51.1	48.9	54.3	40.0	42.9	50.5
RS-Net	44.7	48.4	44.8	49.7	49.6	58.2	47.4	44.8	55.2	59.7	49.3	46.4	51.4	38.6	40.6	48.6
GraphMLP [21]	45.4	50.2	45.8	49.2	51.6	57.9	47.3	44.9	56.9	61.0	49.5	46.9	53.2	37.8	39.9	49.2
DC-GCT [1]	43.3	48.1	44.7	48.2	50.3	**58.2**	45.7	44.8	56.3	62.4	**49.1**	45.4	**52.9**	34.2	**38.9**	48.4
TCSM(Ours)	43.4	47.8	44.3	48.0	48.5	56.8	46.2	44.6	55.0	59.2	48.0	45.5	49.5	36.7	42.0	**47.7**
TCSM (Ours)	42.2	48.3	43.5	48.4	47.9	53.3	46.9	45.2	54.0	55.9	48.5	45.9	48.8	37.0	40.7	**47.1**
P-MPJPE	Dir.	Disc	Eat	Greet	Phone	Photo	Pose	Purch.	Sit	SitD.	Smoke	Wait	WalkD.	Walk	WalkT.	Avg.
FCN [25].	39.5	43.2	46.4	47.0	51.0	56.0	41.4	40.6	56.5	69.4	49.2	45.0	49.5	38.0	43.1	47.7
TCN [29]	36.0	38.7	38.0	41.7	40.1	45.9	37.1	35.4	46.8	53.4	41.4	36.9	43.1	30.3	34.8	40.0
ST-GCN [17]	36.8	38.7	38.2	41.7	40.7	46.8	37.9	35.6	47.6	51.7	41.3	36.8	42.7	31.0	34.7	40.2
SRNet [30]	35.8	39.2	36.6	36.9	39.8	45.1	38.4	36.9	47.7	54.4	38.6	36.3	39.4	30.3	35.4	39.4
MGCN [9]	35.7	38.6	36.3	40.5	39.2	44.5	37.0	35.4	46.4	51.2	40.5	35.6	41.7	30.7	33.9	39.1
SGNN	33.9	37.2	36.8	38.1	38.7	43.5	37.8	35.0	47.2	53.8	40.7	38.3	41.8	30.1	31.4	39.0
RS-Net	35.5	38.3	36.1	40.5	39.2	44.8	37.1	34.9	45.0	49.1	40.2	35.4	41.5	31.0	34.3	38.9
GraphMLP [21]	35.0	38.4	36.6	39.7	40.1	43.9	35.9	34.1	45.9	48.6	40.0	35.3	41.6	30.0	33.3	38.6
DC-GCT [1]	34.4	37.7	35.6	39.5	39.5	44.5	35.9	34.2	44.9	50.1	39.8	34.7	41.5	29.5	32.3	38.3
TCSM(Ours)	33.7	37.1	36.2	38.5	38.2	43.7	35.4	33.8	45.3	48.8	40.8	33.8	40.3	29.6	31.8	**37.8**
TCSM (Ours)	33.4	37.2	35.8	38.3	38.3	43.4	35.6	34.0	44.7	49.0	39.9	34.0	40.5	29.3	32.1	**37.7**

Table 2 further reports the comparison of Params, FLOPS and MPJPE between TCSM and PoseFormer, MixSTE, GraphMLP and DC-GCT with single-frame input. The results show that compared with PoseFormer, our method only requires 44% of the Params while achieving better performance. Compared with DC-GCT, while achieving lower MPJPE, the Params and FLOPs only have a relatively small increment. These results indicate that TCSM achieves competitive performance with less computational cost in 3D human pose estimation from single-frame images.

**Table 2:** Comparison of params, FLOPS and MPJPE on human 3.6M for different single-frame models.

model	Params(M)	FLOPs(M)	MPJPE(mm)↓
PoseFormer [31]	9.56	20	51.3
MixSTE	33.66	1141	51.1
GraphMLP [21]	9.49	348	49.2
DC-GCT [1]	2.07	77	48.4
TCSM(Ours)	4.24	83.49	47.7

### D. Ablation study

To evaluate the effectiveness of the proposed model, we conduct ablation experiments using MPJPE on the Human3.6m dataset.This section studies several hyperparameter combinations to determine the optimal model architecture, as shown in Table 3. In the table, L is the number of TCSM layers, and ${\text{D}}_{\text{a}}$, ${\text{D}}_{\text{G}}$, ${\text{D}}_{\text{M}}$ are the dimensions of the attention channel, GCN channel and MLP channel respectively. With the number of channels unchanged, increasing the number of layers will lead to an increase in the number of parameters. When L increases from 2 to 6, the number of parameters also increases from 3.16 to 7.62, while MPJPE saturates at L = 3 with a value of 47.7 mm. When the number of layers is fixed at 3, we compare different combinations of channel dimensions. It can be seen that when ${\text{D}}_{\text{a}}$ = 128, ${\text{D}}_{\text{G}}$ = 32, ${\text{D}}_{\text{M}}$ = 128, the number of parameters is 4.24M and MPJPE is 47.7 mm, which is the optimal. Therefore, the optimal hyperparameter combination of our model for this task is L = 3, ${\text{D}}_{\text{a}}$ = 128, ${\text{D}}_{\text{G}}$ = 32, ${\text{D}}_{\text{M}}$ = 128.

**Table 3:** Ablation experimental results of hyperparameters for the proposed model.

L	$D_{a}$	$D_{G}$	$D_{M}$	Params(M)	MPJPE(mm)↓
3	128	32	128	4.24	**47.7**
3	128	64	128	4.36	47.9
3	160	64	128	4.44	48.2
3	160	32	128	4.47	48.0
3	160	32	160	4.51	48.4
2	128	32	128	3.14	48.6
4	128	32	128	5.62	48.4
5	128	32	128	6.88	48.5
6	128	32	128	7.62	48.3
3	32	8	32	1.74	49.3
3	64	16	64	2.52	48.7
3	96	24	96	3.46	48.2
3	192	48	192	7.34	48.0

We also study the impact of each component on the model results, as shown in Table 4. In the design without two-stage interactive fusion operation, the pairwise combination of attention channel, GCN channel and MLP channel results in MPJPE always above 49 mm. Although the tri-channel combination design achieves an MPJPE of 48.6 mm, this result is not competitive compared with other state-of-the-art methods. After introducing the two-stage interactive fusion mechanism, each channel first independently learns specialized features, then exchanges key information through the fusion layer, and finally re-optimizes each channel feature with global context, ultimately achieving an MPJPE of 47.7 mm.

**Table 4:** Comparison results of ablation experiments for each component.

model	MPJPE(mm)↓
attn + MLP	49.2
GCN + MLP	49.6
attn + GCN	49.1
attn + GCN + MLP	48.6
attn + GCN + MLP + TCSM	**47.7**

### E. Qualitative analysis

Fig 3 shows the qualitative comparison between TCSM and other state-of-the-art models on the Human3.6M dataset, and our model can accurately predict 3D human poses in indoor environments. To evaluate the generalization performance, Fig 4 shows the visualization results of TCSM on in-the-wild images. Benefiting from the global semantic understanding (Attn), local spatial modeling (GCN) and channel mixing optimization (MLP) of the tri-channel fusion model, our method accurately predicts behaviors significantly different from the Human3.6M training set in most wild scenes. However, as shown in the bottom row of Fig 4, there are still some failure cases caused by severe occlusion, half-body input, excessive 2D detection errors and rare poses.

**Fig 3:**
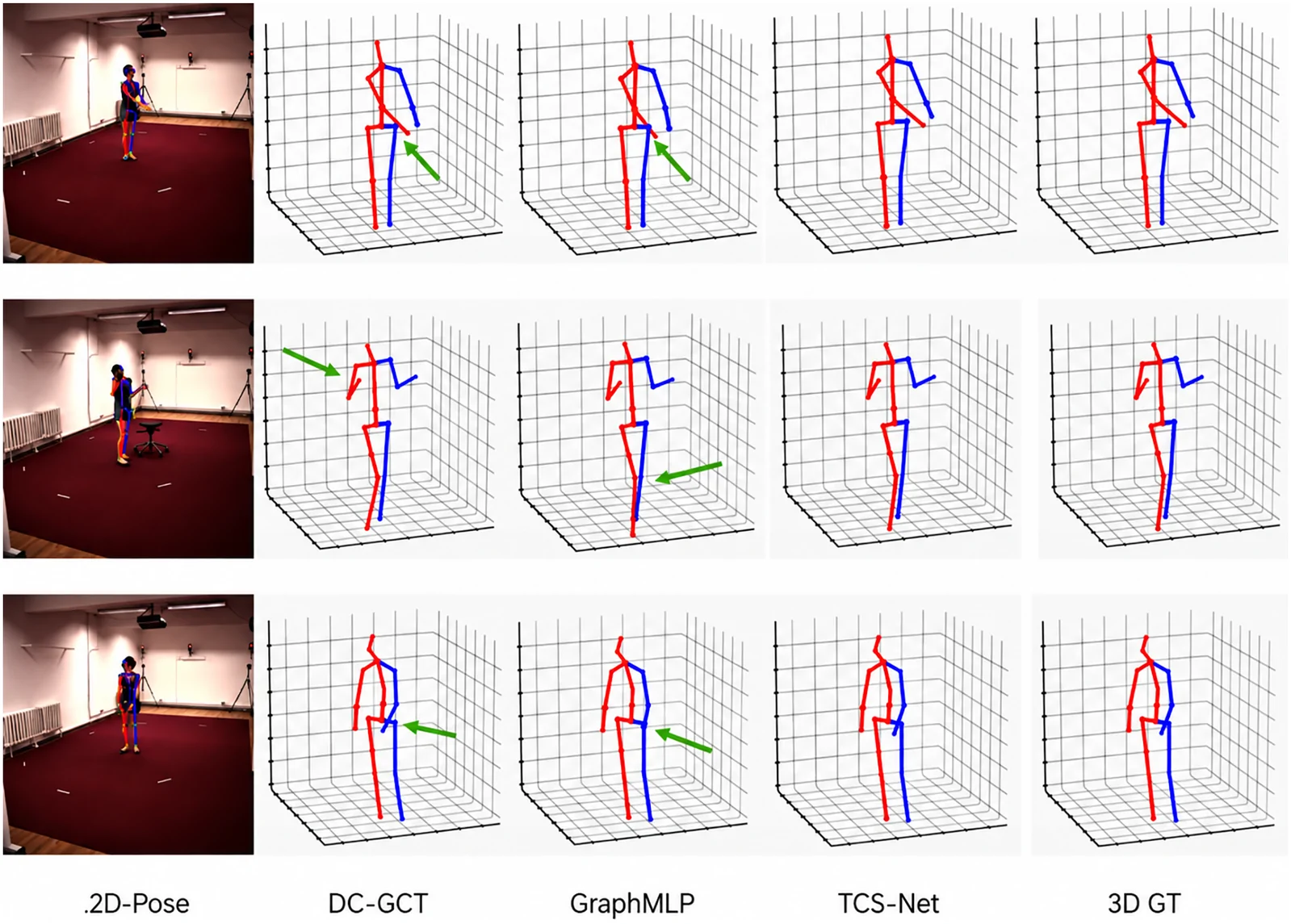
Qualitative comparison on the Human3.6M dataset. Green arrows highlight erroneous estimations.(This figure was created by the authors).

**Fig 4:**
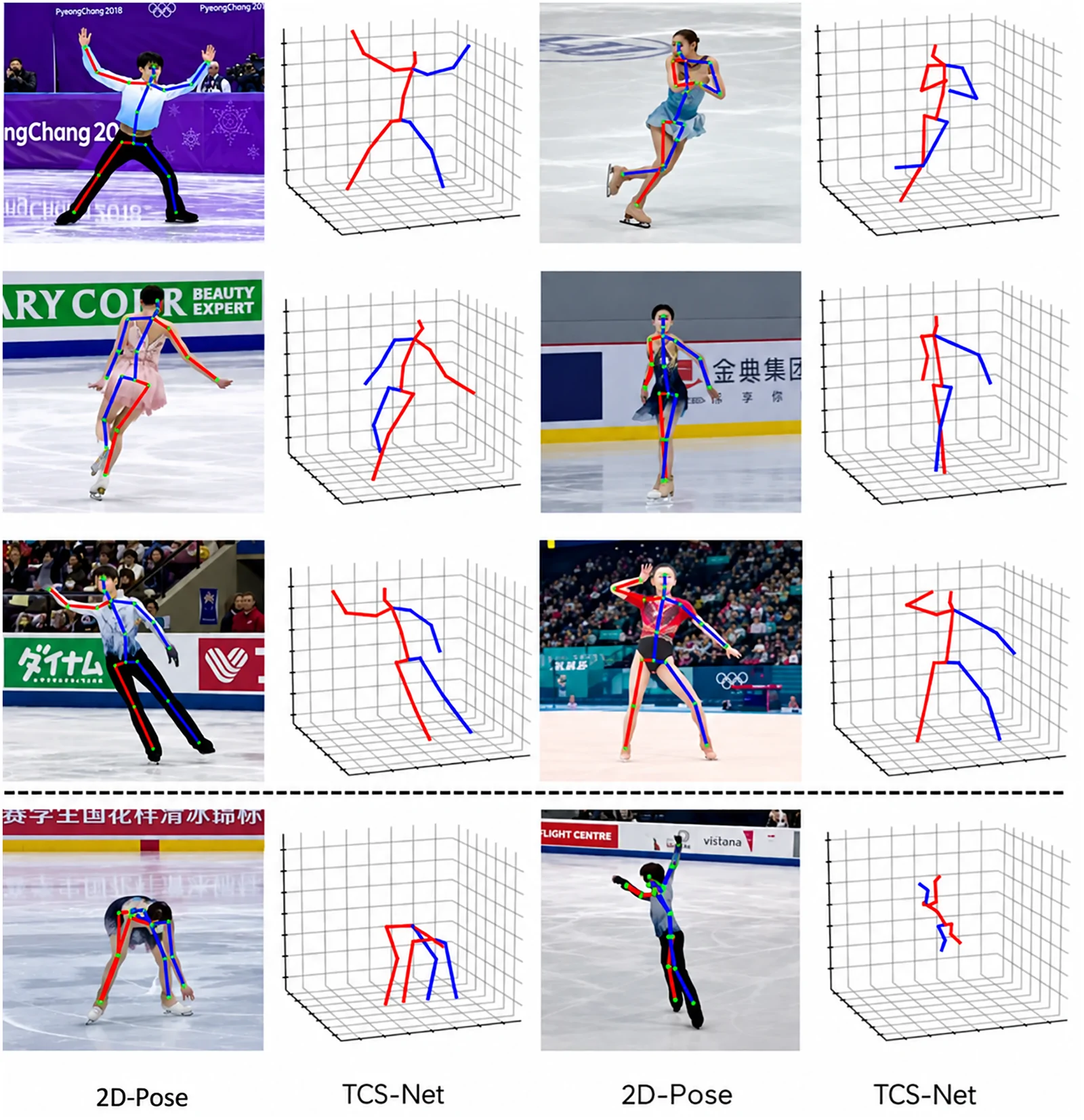
Visualization results of the proposed method on challenging in-the-wild images. The bottom row shows 3D human poses that cannot be accurately generated.(This figure was created by the authors).

## Conclusion

This paper proposes a new model for 3D human pose estimation from monocular images—TCSM. The core of this model lies in its unique tri-channel design, which parallelizes the feature extraction process through three independent but complementary paths: Graph Convolutional Network (GCN) channel, attention mechanism (Attn) channel and Multi-Layer Perceptron (MLP) channel. This parallel structure is specially designed to capture a richer and more diverse set of features from the input 2D pose sequence. The key component of the model is a complex two-stage fusion mechanism that promotes deep information exchange between the three channels, enabling them to complement each other to generate a richer and more comprehensive feature representation.

The core contributions of this research can be summarized into three points. First, the proposed tri-channel parallel architecture provides a powerful and flexible framework for 3D human pose estimation, enabling the model to simultaneously learn local spatial structures, global dependency relationships and complex nonlinear mappings. Second, the two-stage cross-channel fusion mechanism is crucial for realizing the synergistic advantages of the tri-channel design, enabling the model to learn a more comprehensive and accurate final feature representation. Third, extensive experiments based on a widely recognized benchmark dataset show that our TCSM model achieves leading performance, verifying the effectiveness of the proposed model.
