# Supplementary material for: Tri-channel synergistic model for 3D human pose estimation
Source: PLoS One. 2026 Oct 15;21(10):e0359200. doi: 10.1371/journal.pone.0359200 (PMC13671567; doi:10.1371/journal.pone.0359200)
Supplement: Supplementary file 1 [file pone.0359200.s002.docx]

**Manuscript ID:** PONE-D-26-18201

**Title:** A Three-Channel Collaborative Model for 3D Human Pose Estimation

**Dear Academic Editor:**

We sincerely appreciate the editor and the reviewer for their careful evaluation of our manuscript and for providing valuable comments and constructive suggestions. These comments have been very helpful for improving the quality, clarity, and presentation of our work.

We have carefully revised the manuscript according to all the comments. All modifications have been highlighted in the revised manuscript, and corresponding explanations are provided below. For clarity, the reviewer’s comments are presented in bold, followed by our responses.

We would like to express our sincere gratitude for the reviewer’s insightful suggestions.

**1. Please ensure that your manuscript meets PLOS ONE's style requirements, including those for file naming. The PLOS ONE style templates can be found at https://journals.plos.org/plosone/s/file?**

Revision:

The manuscript formatting and file naming have been revised according to the PLOS ONE style requirements.

2. **Please note that PLOS One has specific guidelines on code sharing for submissions in which author-generated code underpins the findings in the manuscript. In these cases, we expect all author-generated code to be made available without restrictions upon publication of the work. Please review our guidelines at https://journals.plos.org/plosone/s/materials-and-software-sharing#loc-sharing-code and ensure that your code is shared in a way that follows best practice and facilitates reproducibility and reuse.**

https://github.com/gy1016-web/Tri-channel

3. **Thank you for stating the following in the Competing Interests section:**

**[The authors have declared that no competing interests exist.].**

**We note that one or more of the authors are employed by a commercial company: Shandong Huayun 3D Technology Co., Ltd.**

Funding Statement: The authors received no specific funding for this work.

Competing Interests Statement: Xinyan Gao is employed by Shandong Huayun 3D Technology Co., Ltd. The company is listed only as the author's institutional affiliation and did not provide funding, equipment, data, materials, or other support for this study. The company had no role in study design, data collection and analysis, decision to publish, or preparation of the manuscript. The authors declare that this commercial affiliation does not alter their adherence to PLOS ONE policies on sharing data, code, and materials. The authors declare that they have no patents, products in development, or marketed products related to this work, and no other competing interests exist.

I have attached the updated funding statement and conflict of interest statement to my cover letter.

4. **PLOS requires an ORCID iD for the corresponding author in Editorial Manager on papers submitted after December 6th, 2016. Please ensure that you have an ORCID iD and that it is validated in Editorial Manager. To do this, go to ‘Update my Information’ (in the upper left-hand corner of the main menu), and click on the Fetch/Validate link next to the ORCID field. This will take you to the ORCID site and allow you to create a new iD or authenticate a pre-existing iD in Editorial Manager.**

The corresponding author has validated the ORCID iD in Editorial Manager.

**5. We note that Figures 1, 3, and 4 in your submission contain images which may be copyrighted. All PLOS content is published under the Creative Commons Attribution License (CC BY 4.0), which means that the manuscript, images, and Supporting Information files will be freely available online, and any third party is permitted to access, download, copy, distribute, and use these materials in any way, even commercially, with proper attribution. For more information, see our copyright guidelines: http://journals.plos.org/plosone/s/licenses-and-copyright.**

Thank you for the reminder regarding figure copyright and licensing. We confirm that Figures 1, 3, and 4 were created by the authors specifically for this study. These figures were not reproduced, adapted, or modified from any previously published or third-party copyrighted material. Therefore, no additional copyright permission is required.

We also confirm that these figures may be published under the Creative Commons Attribution License, CC BY 4.0, together with the article if the manuscript is accepted for publication in PLOS ONE.

**Response to Reviewer**

**Comment 1. Notation inconsistencies and typesetting errors in equations.**

The reviewer noted several notation and typesetting issues, including inconsistent use of j/J in the input dimension, inconsistent notation below Equation 1, an unconventional query transformation matrix in Equation 2, the abrupt appearance of X_BG in Equation 4, and rendering/typesetting glitches in Equations 8 and 9.

Response: Thank you for carefully identifying these notation and typesetting problems. We have thoroughly checked the mathematical expressions and revised the affected equations and explanatory text to improve consistency and readability. Specifically, we made the following corrections:

1) In Section B, we changed the input dimension from a lowercase j to an uppercase J, so that the input tensor notation is consistent with the textual definition of J as the number of joints. The explanatory sentence defining B, J, and C was also retained and clarified. Revision location: Page 10, Lines 194-198.

2) Below Equation 1, we unified the notation for the progressive embedding term and made the symbol in the text consistent with the equation. Revision location: Page 10, Lines 194-198.

3) In Equation 2, we replaced the unconventional query transformation notation with the standard attention projection matrices W_i^Q, W_i^K, and W_i^V, and revised the accompanying explanation to define them as the query, key, and value projection matrices for the i-th attention head. Revision location: Page 11, Lines 221-225.

4) In Equation 4, we corrected the undefined term X_BG to X_gcn according to the variable definitions of the GCN channel. The surrounding explanation of the GCN input feature, adjacency matrix, and learnable weights was also checked. Revision location: Page 12, Lines 242-252.

5) In Equations 8 and 9, we reformatted the split operation and the first-stage channel transformations to remove fragmented or code-like strings and to make the expressions mathematically clear. Revision location: Page 15, Lines 309-314; Page 16-17, Lines 327-3333.

These revisions improve notation consistency and make the mathematical description of the proposed TCSM easier to follow.

**Comment 2. Code Availability.**

While the authors have addressed data availability by pointing out the legal acquisition channels for the public Human3.6M dataset, they should state whether their implementation code, network configurations, and pre-trained weights will be open-sourced to encourage reproducibility in the community.

Response: Thank you for this valuable suggestion. We acknowledge that open code release is vital for result reproduction.The Human3.6M is a well-established public dataset for human pose estimation. The Human3.6M dataset utilized in our experiments is publicly available via the supplied URL. We have constructed a public GitHub repository hosting the TCSM model implementation, relevant scripts, dependency lists and usage tutorials. Upon acceptance and publication, we will upload all network configuration files and trained model weights to this repository or its release page for unrestricted public access.

Human3.6M Dataset:https://drive.google.com/drive/folders/112GPdRC9IEcwcJRyrLJeYw9_YV4wLdKC .

Code repository: https://github.com/gy1016-web/Tri-channel

**Comment 3. Minor grammatical copyediting.**

A few minor phrasing adjustments would polish the manuscript. For example, on line 319, “The key point is that these re-split features...” could be phrased more professionally as “Crucially, these re-split features...”.

Response: Thank you for this helpful suggestion. We carefully proofread the manuscript and revised the relevant sentence to improve the professional tone and readability. In particular, the sentence describing the second-stage feature interaction was rewritten to state that cross-channel information flow allows each second-stage channel to refine its features using complementary information obtained from the other channels in the first stage.

Revision location: Page 16, Lines 317-319.

**Comment 4. Recommended references.**

[1] Peng, Qucheng, Ce Zheng, and Chen Chen. “A dual-augmentor framework for domain generalization in 3D human pose estimation.” In Proceedings of the IEEE/CVF Conference on Computer Vision and Pattern Recognition, pp. 2240-2249. 2024.

[2] Peng, Qucheng, Hongfei Xue, Pu Wang, and Chen Chen. “Lifelong Domain Adaptive 3D Human Pose Estimation.” In Proceedings of the AAAI Conference on Artificial Intelligence, vol. 40, no. 10, pp. 8358-8366. 2026.

Response: Thank you for recommending these recent and relevant studies. We reviewed both publications and found that they are relevant to the broader topic of robustness, domain generalization, and domain adaptation in 3D human pose estimation. We have added them to the revised manuscript and discussed them in the Related Work section. We also clarified the distinction between these studies and our work: the recommended works focus on improving robustness across changing domains, while our TCSM focuses on improving single-frame 2D-to-3D pose lifting through the synergy of GCN, attention, and MLP feature extraction branches.

Revision location: Related Work, Section D, Hybrid Models, Page 7-8, Lines 150-155; References, Page 29, Lines 564-570.

**Final Response**

We sincerely appreciate the Academic Editor and reviewer for their valuable comments and suggestions. These comments have helped us improve the rigor, clarity, reproducibility, and presentation of the manuscript. We hope that the revised manuscript and accompanying materials now satisfy the requirements of PLOS ONE.

Sincerely,

The Authors
