# Supplementary material for: Tri-channel synergistic model for 3D human pose estimation
Source: PLoS One. 2026 Oct 15;21(10):e0359200. doi: 10.1371/journal.pone.0359200 (PMC13671567; doi:10.1371/journal.pone.0359200)
Supplement: Peer review history

# Peer review: Decision Letter 0

**DOI:** 10.1371/journal.pone.0359200.r001

**Authors:** Jason Morgan

7 Jul 2026

Dear Dr. Li,

Thank you for submitting your manuscript to PLOS ONE. After careful consideration, we feel that it has merit but does not fully meet PLOS ONE’s publication criteria as it currently stands. Therefore, we invite you to submit a revised version of the manuscript that addresses the points raised during the review process.

Please submit your revised manuscript by Aug 20 2026 11:59PM. If you will need more time than this to complete your revisions, please reply to this message or contact the journal office at plosone@plos.org. When you're ready to submit your revision, log on to https://www.editorialmanager.com/pone/ and select the 'Submissions Needing Revision' folder to locate your manuscript file.

- A letter that responds to each point raised by the academic editor and reviewer(s). You should upload this letter as a separate file labeled 'Response to Reviewers'.
- A marked-up copy of your manuscript that highlights changes made to the original version. You should upload this as a separate file labeled 'Revised Manuscript with Track Changes'.
- An unmarked version of your revised paper without tracked changes. You should upload this as a separate file labeled 'Manuscript'.

If you would like to make changes to your financial disclosure, please include your updated statement in your cover letter. Guidelines for resubmitting your figure files are available below the reviewer comments at the end of this letter.

If applicable, we recommend that you deposit your laboratory protocols in protocols.io to enhance the reproducibility of your results. Protocols.io assigns your protocol its own identifier (DOI) so that it can be cited independently in the future. For instructions see: https://journals.plos.org/plosone/s/submission-guidelines#loc-laboratory-protocols. Additionally, PLOS ONE offers an option for publishing peer-reviewed Lab Protocol articles, which describe protocols hosted on protocols.io. Read more information on sharing protocols at https://plos.org/protocols?utm_medium=editorial-email&utm_source=authorletters&utm_campaign=protocols.

As the corresponding author, your ORCID iD is verified in the submission system and will appear in the published article. PLOS supports the use of ORCID, and we encourage all coauthors to register for an ORCID iD and use it as well. Please encourage your coauthors to verify their ORCID iD within the submission system before final acceptance, as unverified ORCID iDs will not appear in the published article. *Only*  the individual author can complete the verification step; PLOS staff *cannot*  verify ORCID iDs on behalf of authors.

We look forward to receiving your revised manuscript.

Kind regards,

Dr Jason Morgan

Staff Editor

PLOS One

Journal Requirements:

When submitting your revision, we need you to address these additional requirements.

1. Please ensure that your manuscript meets PLOS ONE's style requirements, including those for file naming. The PLOS ONE style templates can be found at https://journals.plos.org/plosone/s/file?id=wjVg/PLOSOne_formatting_sample_main_body.pdf and https://journals.plos.org/plosone/s/file?id=ba62/PLOSOne_formatting_sample_title_authors_affiliations.pdf

2. Please note that PLOS One has specific guidelines on code sharing for submissions in which author-generated code underpins the findings in the manuscript. In these cases, we expect all author-generated code to be made available without restrictions upon publication of the work. Please review our guidelines at https://journals.plos.org/plosone/s/materials-and-software-sharing#loc-sharing-code and ensure that your code is shared in a way that follows best practice and facilitates reproducibility and reuse.

3. Thank you for stating the following in the Competing Interests section:

[The authors have declared that no competing interests exist.].

We note that one or more of the authors are employed by a commercial company: Shandong Huayun 3D Technology Co., Ltd.

1. Please provide an amended Funding Statement declaring this commercial affiliation, as well as a statement regarding the Role of Funders in your study. If the funding organization did not play a role in the study design, data collection and analysis, decision to publish, or preparation of the manuscript and only provided financial support in the form of authors' salaries and/or research materials, please review your statements relating to the author contributions, and ensure you have specifically and accurately indicated the role(s) that these authors had in your study. You can update author roles in the Author Contributions section of the online submission form.

Please also include the following statement within your amended Funding Statement.

“The funder provided support in the form of salaries for authors [insert relevant initials], but did not have any additional role in the study design, data collection and analysis, decision to publish, or preparation of the manuscript. The specific roles of these authors are articulated in the ‘author contributions’ section.”

If your commercial affiliation did play a role in your study, please state and explain this role within your updated Funding Statement.

2. Please also provide an updated Competing Interests Statement declaring this commercial affiliation along with any other relevant declarations relating to employment, consultancy, patents, products in development, or marketed products, etc.

Within your Competing Interests Statement, please confirm that this commercial affiliation does not alter your adherence to all PLOS ONE policies on sharing data and materials by including the following statement: "This does not alter our adherence to PLOS ONE policies on sharing data and materials.” (as detailed online in our guide for authors http://journals.plos.org/plosone/s/competing-interests) . If this adherence statement is not accurate and there are restrictions on sharing of data and/or materials, please state these. Please note that we cannot proceed with consideration of your article until this information has been declared.

Please include both an updated Funding Statement and Competing Interests Statement in your cover letter. We will change the online submission form on your behalf.

4. PLOS requires an ORCID iD for the corresponding author in Editorial Manager on papers submitted after December 6th, 2016. Please ensure that you have an ORCID iD and that it is validated in Editorial Manager. To do this, go to ‘Update my Information’ (in the upper left-hand corner of the main menu), and click on the Fetch/Validate link next to the ORCID field. This will take you to the ORCID site and allow you to create a new iD or authenticate a pre-existing iD in Editorial Manager.

5. We note that Figures 1, 3, and 4 in your submission contain images which may be copyrighted. All PLOS content is published under the Creative Commons Attribution License (CC BY 4.0), which means that the manuscript, images, and Supporting Information files will be freely available online, and any third party is permitted to access, download, copy, distribute, and use these materials in any way, even commercially, with proper attribution. For more information, see our copyright guidelines: http://journals.plos.org/plosone/s/licenses-and-copyright.

We require you to either (1) present written permission from the copyright holder to publish these figures specifically under the CC BY 4.0 license, or (2) remove the figures from your submission:

1. You may seek permission from the original copyright holder of Figures 1, 3, and 4 to publish the content specifically under the CC BY 4.0 license.

We recommend that you contact the original copyright holder with the Content Permission Form (http://journals.plos.org/plosone/s/file?id=7c09/content-permission-form.pdf) and the following text:

“I request permission for the open-access journal PLOS ONE to publish XXX under the Creative Commons Attribution License (CCAL) CC BY 4.0 (http://creativecommons.org/licenses/by/4.0/). Please be aware that this license allows unrestricted use and distribution, even commercially, by third parties. Please reply and provide explicit written permission to publish XXX under a CC BY license and complete the attached form.”

Please upload the completed Content Permission Form or other proof of granted permissions as an "Other" file with your submission.

In the figure caption of the copyrighted figure, please include the following text: “Reprinted from [ref] under a CC BY license, with permission from [name of publisher], original copyright [original copyright year].”

2. If you are unable to obtain permission from the original copyright holder to publish these figures under the CC BY 4.0 license or if the copyright holder’s requirements are incompatible with the CC BY 4.0 license, please either i) remove the figure or ii) supply a replacement figure that complies with the CC BY 4.0 license. Please check copyright information on all replacement figures and update the figure caption with source information. If applicable, please specify in the figure caption text when a figure is similar but not identical to the original image and is therefore for illustrative purposes only.

If the reviewer comments include a recommendation to cite specific previously published works, please review and evaluate these publications to determine whether they are relevant and should be cited. There is no requirement to cite these works unless the editor has indicated otherwise.

[Note: HTML markup is below. Please do not edit.]

Reviewers' comments:

Reviewer's Responses to Questions

**Comments to the Author**

1. Is the manuscript technically sound, and do the data support the conclusions?

Reviewer #1: Yes

**********

2. Has the statistical analysis been performed appropriately and rigorously?

Reviewer #1: Yes

**********

3. Have the authors made all data underlying the findings in their manuscript fully available?

The PLOS Data policy

Reviewer #1: Yes

**********

4. Is the manuscript presented in an intelligible fashion and written in standard English?

Reviewer #1: Yes

**********

Reviewer #1: Review Report on PONE-D-26-18201: Tri-Channel Synergistic Model for 3D Human Pose Estimation

Summary of the Manuscript:

This paper proposes a novel Tri-Channel Synergistic Model (TCSM) designed for 3D human pose estimation from single-view monocular images. The core concept introduces a parallel architecture that decouples structural dependencies using three distinct computational paradigms: a Graph Convolutional Network (GCN) channel for topological physical constraints, a self-attention mechanism channel for global long-range correlations, and a Multi-Layer Perceptron (MLP) channel for efficient global context encoding. To foster deep interaction between these streams, the authors introduce a two-stage feature fusion and refinement mechanism. Evaluated on the widely recognized Human3.6M dataset, the method demonstrates superior performance (47.7mm MPJPE and 37.8mm P-MPJPE) relative to state-of-the-art single-frame alternatives.

Major Strengths:

1. Well-Motivated Architecture: The motivation to systematically decouple and synergize the heterogeneous nature of human pose features (structural, dynamic correlation, and global context) into independent parallel streams is sound and fills a clear gap in existing single- and dual-stream network designs.

2. Strong Experimental Evaluation: The model is validated on an authoritative baseline benchmark dataset (Human3.6M). It achieves highly competitive results against prominent architectures like PoseFormer, GraphMLP, and DC-GCT, while optimizing parameter efficiency.

3. Detailed Ablation Studies: The comprehensive ablation experiments regarding the number of layers, channel dimensions, and the necessity of the two-stage interactive fusion module cleanly substantiate the design choices.

Minor Weaknesses and Areas for Improvement:

While the manuscript is technically sound and well-written, the following minor points and notation errors should be addressed in a minor revision:

1. Notation Inconsistencies and Typesetting Errors in Equations:

- In Section B (Page 9), the input dimensions are defined using a lowercase "j" ($X_{in}\in\mathbb{R}^{B\times j\times2}$), whereas the text directly below defines "J" (uppercase) as the number of joints. Please make this consistent.

- Below Equation 1, the text references $\epsilon_{prog}(\cdot)$, but Equation 1 itself writes it as $E_{prog}(\cdot)$.

- In Equation 2 (Page 10), the Query transformation matrix is written using an unconventional symbol ($\mathbb{W}_{i}^{\Omega}$) instead of a standard $W_i^Q$ notation, which contradicts the text indicating Query (Q).

- In Equation 4 (Page 12), the term $X_{BGi}$ appears abruptly inside the summation. Based on the variable definitions below, this seems to be a typographical error for $X_{gcn}$.

- In Section C.4 (Page 15), Equations 8 and 9 contain prominent rendering/typesetting glitches. For instance, Equation 8 uses an awkward comma placement ($X_{g'}X_{a},X_{m}$), and Equation 9 contains fragmented text strings ("(X) = GCN_Channel(X)", "Ag", "=", "Attention_Channel(Xa)"). These mathematical expressions must be cleanly reformatted.

2. Code Availability:

- While the authors have addressed data availability by pointing out the legal acquisition channels for the public Human3.6M dataset, they should state whether their implementation code, network configurations, and pre-trained weights will be open-sourced to encourage reproducibility in the community.

3. Minor Grammatical Copyediting:

- A few minor phrasing adjustments would polish the manuscript. For example, on line 319, "The key point is that these re-split features..." could be phrased more professionally as "Crucially, these re-split features...".

4. Recommended References:

[1] Peng, Qucheng, Ce Zheng, and Chen Chen. "A dual-augmentor framework for domain generalization in 3d human pose estimation." In Proceedings of the IEEE/CVF Conference on Computer Vision and Pattern Recognition, pp. 2240-2249. 2024.

[2] Peng, Qucheng, Hongfei Xue, Pu Wang, and Chen Chen. "Lifelong Domain Adaptive 3D Human Pose Estimation." In Proceedings of the AAAI Conference on Artificial Intelligence, vol. 40, no. 10, pp. 8358-8366. 2026.

Recommendation:

Minor Revision. The technical contributions are solid, and fixing the mathematical notations and clarifying code availability will bring the manuscript to a highly publishable standard.

**********

what does this mean?). If published, this will include your full peer review and any attached files.

If you choose “no”, your identity will remain anonymous but your review may still be made public.

**Do you want your identity to be public for this peer review?**  For information about this choice, including consent withdrawal, please see our Privacy Policy

Reviewer #1: No

**********

[NOTE: If reviewer comments were submitted as an attachment file, they will be attached to this email and accessible via the submission site. Please log into your account, locate the manuscript record, and check for the action link "View Attachments". If this link does not appear, there are no attachment files.]

To ensure your figures meet our technical requirements, please review our figure guidelines: https://journals.plos.org/plosone/s/figures

You may also use PLOS’s free figure tool, NAAS, to help you prepare publication quality figures: https://journals.plos.org/plosone/s/figures#loc-tools-for-figure-preparation.

NAAS will assess whether your figures meet our technical requirements by comparing each figure against our figure specifications.

# Author comment: Author response to Decision Letter 1

**DOI:** 10.1371/journal.pone.0359200.r002

17 Jul 2026

Dear Academic Editor:

We sincerely appreciate the editor and the reviewer for their careful evaluation of our manuscript and for providing valuable comments and constructive suggestions. These comments have been very helpful for improving the quality, clarity, and presentation of our work.

We have carefully revised the manuscript according to all the comments. All modifications have been highlighted in the revised manuscript, and corresponding explanations are provided below. For clarity, the reviewer’s comments are presented in bold, followed by our responses.

We would like to express our sincere gratitude for the reviewer’s insightful suggestions.

1. Please ensure that your manuscript meets PLOS ONE's style requirements, including those for file naming. The PLOS ONE style templates can be found at https://journals.plos.org/plosone/s/file?

Revision:

The manuscript formatting and file naming have been revised according to the PLOS ONE style requirements.

2.Please note that PLOS One has specific guidelines on code sharing for submissions in which author-generated code underpins the findings in the manuscript. In these cases, we expect all author-generated code to be made available without restrictions upon publication of the work. Please review our guidelines at https://journals.plos.org/plosone/s/materials-and-software-sharing#loc-sharing-code and ensure that your code is shared in a way that follows best practice and facilitates reproducibility and reuse.

https://github.com/gy1016-web/Tri-channel

3.Thank you for stating the following in the Competing Interests section:

[The authors have declared that no competing interests exist.].

We note that one or more of the authors are employed by a commercial company: Shandong Huayun 3D Technology Co., Ltd.

Funding Statement: The authors received no specific funding for this work.

Competing Interests Statement: Xinyan Gao is employed by Shandong Huayun 3D Technology Co., Ltd. The company is listed only as the author's institutional affiliation and did not provide funding, equipment, data, materials, or other support for this study. The company had no role in study design, data collection and analysis, decision to publish, or preparation of the manuscript. The authors declare that this commercial affiliation does not alter their adherence to PLOS ONE policies on sharing data, code, and materials. The authors declare that they have no patents, products in development, or marketed products related to this work, and no other competing interests exist.

I have attached the updated funding statement and conflict of interest statement to my cover letter.

4.PLOS requires an ORCID iD for the corresponding author in Editorial Manager on papers submitted after December 6th, 2016. Please ensure that you have an ORCID iD and that it is validated in Editorial Manager. To do this, go to ‘Update my Information’ (in the upper left-hand corner of the main menu), and click on the Fetch/Validate link next to the ORCID field. This will take you to the ORCID site and allow you to create a new iD or authenticate a pre-existing iD in Editorial Manager.

The corresponding author has validated the ORCID iD in Editorial Manager.

5. We note that Figures 1, 3, and 4 in your submission contain images which may be copyrighted. All PLOS content is published under the Creative Commons Attribution License (CC BY 4.0), which means that the manuscript, images, and Supporting Information files will be freely available online, and any third party is permitted to access, download, copy, distribute, and use these materials in any way, even commercially, with proper attribution. For more information, see our copyright guidelines: http://journals.plos.org/plosone/s/licenses-and-copyright.

Thank you for the reminder regarding figure copyright and licensing. We confirm that Figures 1, 3, and 4 were created by the authors specifically for this study. These figures were not reproduced, adapted, or modified from any previously published or third-party copyrighted material. Therefore, no additional copyright permission is required.

We also confirm that these figures may be published under the Creative Commons Attribution License, CC BY 4.0, together with the article if the manuscript is accepted for publication in PLOS ONE.

Response to Reviewer

Comment 1. Notation inconsistencies and typesetting errors in equations.

The reviewer noted several notation and typesetting issues, including inconsistent use of j/J in the input dimension, inconsistent notation below Equation 1, an unconventional query transformation matrix in Equation 2, the abrupt appearance of X_BG in Equation 4, and rendering/typesetting glitches in Equations 8 and 9.

Response: Thank you for carefully identifying these notation and typesetting problems. We have thoroughly checked the mathematical expressions and revised the affected equations and explanatory text to improve consistency and readability. Specifically, we made the following corrections:

1) In Section B, we changed the input dimension from a lowercase j to an uppercase J, so that the input tensor notation is consistent with the textual definition of J as the number of joints. The explanatory sentence defining B, J, and C was also retained and clarified. Revision location: Page 10, Lines 194-198.

2) Below Equation 1, we unified the notation for the progressive embedding term and made the symbol in the text consistent with the equation. Revision location: Page 10, Lines 194-198.

3) In Equation 2, we replaced the unconventional query transformation notation with the standard attention projection matrices W_i^Q, W_i^K, and W_i^V, and revised the accompanying explanation to define them as the query, key, and value projection matrices for the i-th attention head. Revision location: Page 11, Lines 221-225.

4) In Equation 4, we corrected the undefined term X_BG to X_gcn according to the variable definitions of the GCN channel. The surrounding explanation of the GCN input feature, adjacency matrix, and learnable weights was also checked. Revision location: Page 12, Lines 242-252.

5) In Equations 8 and 9, we reformatted the split operation and the first-stage channel transformations to remove fragmented or code-like strings and to make the expressions mathematically clear. Revision location: Page 15, Lines 309-314; Page 16-17, Lines 327-3333.

These revisions improve notation consistency and make the mathematical description of the proposed TCSM easier to follow.

Comment 2. Code Availability.

While the authors have addressed data availability by pointing out the legal acquisition channels for the public Human3.6M dataset, they should state whether their implementation code, network configurations, and pre-trained weights will be open-sourced to encourage reproducibility in the community.

Response: Thank you for this valuable suggestion. We acknowledge that open code release is vital for result reproduction.The Human3.6M is a well-established public dataset for human pose estimation. The Human3.6M dataset utilized in our experiments is publicly available via the supplied URL. We have constructed a public GitHub repository hosting the TCSM model implementation, relevant scripts, dependency lists and usage tutorials. Upon acceptance and publication, we will upload all network configuration files and trained model weights to this repository or its release page for unrestricted public access.

Human3.6M Dataset:https://drive.google.com/drive/folders/112GPdRC9IEcwcJRyrLJeYw9_YV4wLdKC .

Code repository: https://github.com/gy1016-web/Tri-channel

Comment 3. Minor grammatical copyediting.

A few minor phrasing adjustments would polish the manuscript. For example, on line 319, “The key point is that these re-split features...” could be phrased more professionally as “Crucially, these re-split features...”.

Response: Thank you for this helpful suggestion. We carefully proofread the manuscript and revised the relevant sentence to improve the professional tone and readability. In particular, the sentence describing the second-stage feature interaction was rewritten to state that cross-channel information flow allows each second-stage channel to refine its features using complementary information obtained from the other channels in the first stage.

Revision location: Page 16, Lines 317-319.

Comment 4. Recommended references.

[1] Peng, Qucheng, Ce Zheng, and Chen Chen. “A dual-augmentor framework for domain generalization in 3D human pose estimation.” In Proceedings of the IEEE/CVF Conference on Computer Vision and Pattern Recognition, pp. 2240-2249. 2024.

[2] Peng, Qucheng, Hongfei Xue, Pu Wang, and Chen Chen. “Lifelong Domain Adaptive 3D Human Pose Estimation.” In Proceedings of the AAAI Conference on Artificial Intelligence, vol. 40, no. 10, pp. 8358-8366. 2026.

Response: Thank you for recommending these recent and relevant studies. We reviewed both publications and found that they are relevant to the broader topic of robustness, domain generalization, and domain adaptation in 3D human pose estimation. We have added them to the revised manuscript and discussed them in the Related Work section. We also clarified the distinction between these studies and our work: the recommended works focus on improving robustness across changing domains, while our TCSM focuses on improving single-frame 2D-to-3D pose lifting through the synergy of GCN, attention, and MLP feature extraction branches.

Revision location: Related Work, Section D, Hybrid Models, Page 7-8, Lines 150-155; References, Page 29, Lines 564-570.

Final Response

We sincerely appreciate the Academic Editor and reviewer for their valuable comments and suggestions. These comments have helped us improve the rigor, clarity, reproducibility, and presentation of the manuscript. We hope that the revised manuscript and accompanying materials now satisfy the requirements of PLOS ONE.

Sincerely,

The Authors

Attachment Submitted filename: Response to Reviewers.docx

# Peer review: Decision Letter 1

**DOI:** 10.1371/journal.pone.0359200.r003

**Authors:** Feng Ding

10 Sep 2026

Tri-Channel Synergistic Model for 3D Human Pose Estimation

PONE-D-26-18201R1

Dear Dr. Li,

We’re pleased to inform you that your manuscript has been judged scientifically suitable for publication and will be formally accepted for publication once it meets all outstanding technical requirements.

Within one week, you’ll receive an e-mail detailing the required amendments. When these have been addressed, you’ll receive a formal acceptance letter and your manuscript will be scheduled for publication.

An invoice will be generated when your article is formally accepted. Please note, if your institution has a publishing partnership with PLOS and your article meets the relevant criteria, all or part of your publication costs will be covered. Please make sure your user information is up-to-date by logging into Editorial Manager at Editorial Manager® and clicking the ‘Update My Information' link at the top of the page. For questions related to billing, please contact billing support.

If your institution or institutions have a press office, please notify them about your upcoming paper to help maximize its impact. If they’ll be preparing press materials, please inform our press team as soon as possible -- no later than 48 hours after receiving the formal acceptance. Your manuscript will remain under strict press embargo until 2 pm Eastern Time on the date of publication. For more information, please contact onepress@plos.org.

Kind regards,

Feng Ding

Academic Editor

PLOS One

Additional Editor Comments (optional):

Reviewers' comments:

Reviewer's Responses to Questions

**Comments to the Author**

Reviewer #1: All comments have been addressed

Reviewer #2: All comments have been addressed

**********

2. Is the manuscript technically sound, and do the data support the conclusions?

Reviewer #1: Yes

Reviewer #2: Yes

**********

3. Has the statistical analysis been performed appropriately and rigorously?

Reviewer #1: Yes

Reviewer #2: Yes

**********

4. Have the authors made all data underlying the findings in their manuscript fully available?

The PLOS Data policy

Reviewer #1: Yes

Reviewer #2: Yes

**********

5. Is the manuscript presented in an intelligible fashion and written in standard English?

Reviewer #1: Yes

Reviewer #2: Yes

**********

Reviewer #1: Thank the authors for their comprehensive explanations and modifications! My concerns have been addressed completely and I do not have further comments. I recommend that this paper should be accepted.

Reviewer #2: Having reviewed the paper thoroughly, I have no further remarks and am pleased to accept it for publication.

**********

what does this mean?). If published, this will include your full peer review and any attached files.

If you choose “no”, your identity will remain anonymous but your review may still be made public.

**Do you want your identity to be public for this peer review?**  For information about this choice, including consent withdrawal, please see our Privacy Policy

Reviewer #1: No

Reviewer #2: **Yes:**  Yushan Pan

**********

# Editor report: Acceptance letter

**DOI:** 10.1371/journal.pone.0359200.r004

**Authors:** Feng Ding

PONE-D-26-18201R1

PLOS One

Dear Dr. Li,

I'm pleased to inform you that your manuscript has been deemed suitable for publication in PLOS One. Congratulations! Your manuscript is now being handed over to our production team.

At this stage, our production department will prepare your paper for publication. This includes ensuring the following:

* All references, tables, and figures are properly cited

* All relevant supporting information is included in the manuscript submission,

* There are no issues that prevent the paper from being properly typeset

You will receive further instructions from the production team, including instructions on how to review your proof when it is ready. Please keep in mind that we are working through a large volume of accepted articles, so please give us a few days to review your paper and let you know the next and final steps.

Lastly, if your institution or institutions have a press office, please let them know about your upcoming paper now to help maximize its impact. If they'll be preparing press materials, please inform our press team within the next 48 hours. Your manuscript will remain under strict press embargo until 2 pm Eastern Time on the date of publication. For more information, please contact onepress@plos.org.

You will receive an invoice from PLOS for your publication fee after your manuscript has reached the completed accept phase. If you receive an email requesting payment before acceptance or for any other service, this may be a phishing scheme. Learn how to identify phishing emails and protect your accounts at https://explore.plos.org/phishing.

If we can help with anything else, please email us at customercare@plos.org.

Thank you for submitting your work to PLOS One and supporting open access.

Kind regards,

PLOS One Editorial Office Staff

on behalf of

Dr. Feng Ding

Academic Editor

PLOS One
